# Increased Pneumonia-Related Emergency Department Visits, Northern Italy

**DOI:** 10.3201/eid3105.241790

**Published:** 2025-05

**Authors:** Simone Villa, Manuel Maffeo, Mauro Maistrello, Giorgio Bagarella, Vincenzo Navobi Porrello, Federica Morani, Francesco Scovenna, Fausto Baldanti, Paolo Bonfanti, Elena Pariani, Andrea Gori, Luigi Vezzosi, Gabriele Del Castillo, Sabrina Buoro, Danilo Cereda

**Affiliations:** University of Milan, Milan, Italy (S. Villa, F. Scovenna, E. Pariani, A. Gori); Directorate General for Health of the Lombardy Region, Milan (M. Maffeo, M. Maistrello, G. Bagarella, L. Vezzosi, G. Del Castillo, S. Buoro, D. Cereda); University of Pavia, Pavia, Italy (V.N. Porrello, F. Baldanti); Istituto di Ricovero e Cura a Carattere Scientifico San Raffaele Scientific Institute, Milan (F. Morani); Fondazione Istituto di Ricovero e Cura a Carattere Scientifico Policlinico San Matteo, Pavia (F. Baldanti); University of Milano Bicocca, Monza, Italy (P. Bonfanti)

**Keywords:** pneumonia, bacteria, syndromic surveillance, *Mycoplasma pneumoniae*, *Bordetella pertussis*, outbreak, Italy

## Abstract

An increase in pneumonia-related emergency department visits was observed in Lombardy, northern Italy, during June–October 2024. Viral causes appear insufficient to explain the increase, suggesting a bacterial cause. *Mycoplasma pneumoniae* and *Bordetella pertussis* emerged as possible causes when other surveillance systems were consulted, but the reasons behind this trend remain unknown.

The Emergency Department (ED) Syndromic Surveillance (EDSyS) system has been implemented in various settings and has proven to be useful for detecting early signs of infectious disease outbreaks (*1*). In preparation for the 2024–25 influenza season and to enhance pandemic preparedness, EDSyS was implemented across the Lombardy region (≈10 million population) in northern Italy, covering all 103 EDs. This system can provide early warnings of public health issues and to adapt health care capacity in response to seasonal public health challenges such as influenza and emergent or unknown threats. Visits are classified by diagnosis and coded by using the International Classification of Diseases, 9th Revision. Visits were coded according to Centers for Disease Control and Prevention guidelines (*2*), including COVID-19 pneumonia, and were classified as pneumonia-associated. Those visits were monitored and analyzed against 2021–2023 figures, disaggregated by age groups (<1 year, 1–4 years, 5–17 years, 18–64 years, and *>*65 years). The data in this article were last updated on November 4, 2024.

Total ED visits (Appendix Figure 1) were constantly elevated throughout 2024, most notable in summer during July (week 27 total 70,599 vs. historic maximum of 67,835) and August (week 35 total 67,540 vs. historic maximum of 60,710). Concurrently, a considerable increase in pneumonia-associated visits was recorded from week 18 of 1,164 visits, compared with the previous maximum of 959 visits in 2021 (Figure 1), largely among those 5–17 years of age.

**Figure 1:**
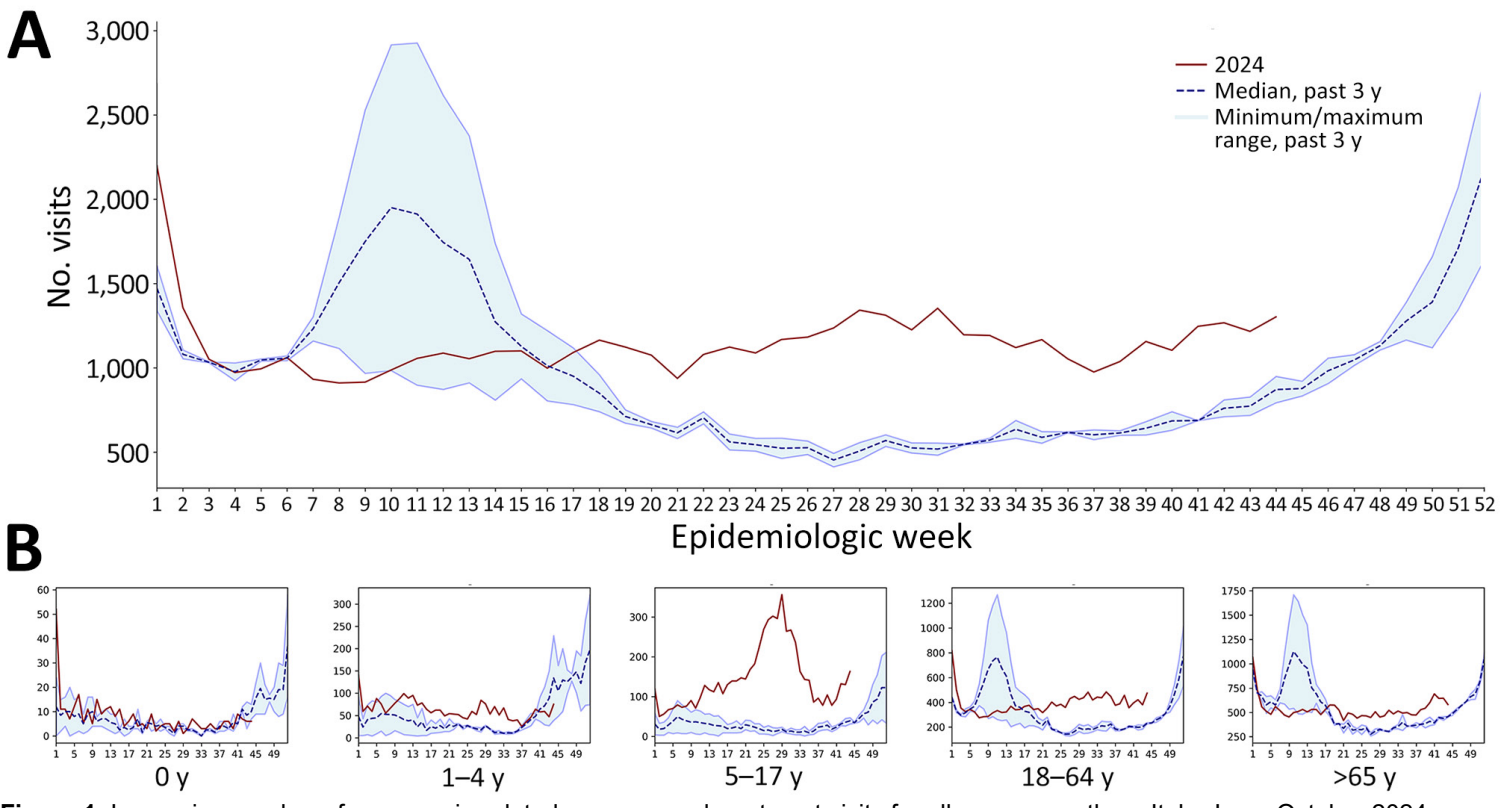
Increasing number of pneumonia-related emergency department visits for all causes, northern Italy, June–October 2024, compared with previous 3 years. A) Weekly number of pneumonia-associated emergency department visits across all patients. B) Weekly number of pneumonia-associated emergency department visits by age group.

During June–September 2024, there were 10,022 pneumonia-associated hospitalizations compared with 8,118 in 2023. Most hospitalizations were coded as pneumonia, organism unspecified (3,602 cases), bacterial pneumonia, unspecified (1,906 cases), or bronchopneumonia, unspecified organism (1,515 cases). Compared with the summer of 2023, a marked increase was observed for pneumonia caused by *Mycoplasma pneumoniae* (769 cases in 2024 vs. 53 cases in 2023, a 14.5-fold increase). Likewise, 85 cases of pneumonia caused by *Chlamydophila pneumoniae* were recorded in the summer 2024 versus 33 cases in 2023 (2.6-fold increase). In addition, 3 cases of pneumonia caused by *Bordetella pertussi* were reported in the summer of 2024 summer compared with none in 2023.

Because of our findings, we reviewed data from 3 other regional surveillance systems, focusing on the notification of infectious diseases, community syndromic surveillance, and virologic surveillance of respiratory viruses in EDs, to identify trends or outbreaks of causes of pneumonia that may have gone unnoticed. Our search focused on diseases reported through the statutory regional notification system for infectious diseases, including pneumonia caused by *M. pneumoniae* as a mandatory notification at the regional level since December 2023. In total, 366 notifications of pneumonia caused by *M. pneumoniae* were recorded, and high weekly frequency occurred during week 18 (April 29, 2024) through week 45 (November 4, 2024). Notifications of pneumonia caused by *M. pneumoniae* peaked in week 29 (July 15, 2024), when 24 cases were reported (Figure 2, panel A), mostly in hospitalized persons (72%, n = 263) and in persons 5–17 years old (52%, n = 189). For pertussis, 180 cases were recorded in 2024 (vs. 4 in 2023; 44-fold increase), mostly in persons <1 year of age (23%, n = 42) and 5–17 years of age (46%, n = 83). Notifications of pertussis reached peaks in June (30 cases) and July (34 cases) (Figure 2, panel B).

**Figure 2:**
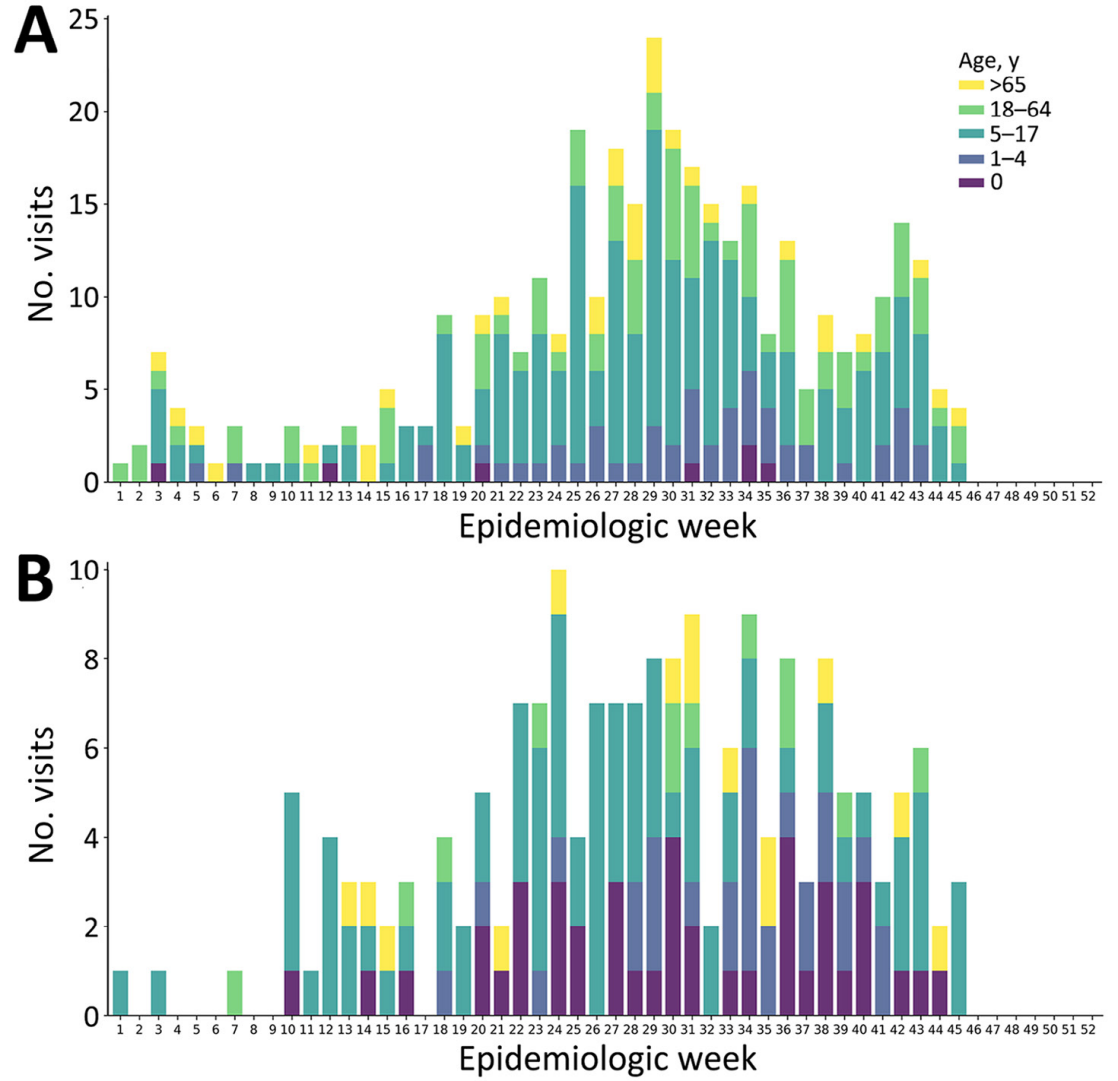
Increasing number of pneumonia-related emergency department visits for *Mycoplasma pneumoniae* (A) and pertussis (B), by age group, northern Italy, June–October 2024. Cases were notified by the regional infectious disease surveillance systems.

The surveillance system for influenza-like illness (ILI), part of the surveillance network (RespiVirNet, https://www.iss.it/en/respivirnet) of Italy, which uses data reported by general practitioners and pediatricians, was unable to identify any major change in ILI incidence (Appendix Figure 2). Likewise, existing virologic surveillance of ILI-associated ED visits during summer 2024 did not detect increased activity of any viral pathogens.

Because of the stringent measures implemented to curb the increase of COVID-19 cases in 2020, the population was less exposed to periodic small epidemics of common respiratory infections (*3*), and immunization programs were slowed down or halted, including immunization of pregnant women and children against pertussis (*4*). Other countries observed increased cases of pneumonia caused by *M. pneumoniae*, starting at the end of 2023, when China reported clusters of respiratory diseases in children (*5*). More recently, the United Kingdom (*6*) and the United States (*7*) also reported increased cases of those causes of pneumonia. Likewise, after a decline in cases of pertussis was observed during the COVID-19 pandemic, the disease returned to prepandemic levels or higher, as reported by some countries (*8*). Despite no decrease in vaccination coverage, an immunity debt might be the underlying reason for the increase in cases (*9*). Other pathogens, or a combination of pathogens, might be responsible for the observed increase in pneumonia-associated ED visits (*10*).

In conclusion, in northern Italy, we are extending ED surveillance to include bacteria such as *M. pneumoniae* and *B. pertussis*. We have found the newly implemented EDSyS is a promising tool for early detection of infectious diseases outbreaks; additional improvements to the system are in development. In particular, the range of syndromes covered will be expanded to include gastrointestinal syndromes, and automatic alerts for outbreak detection will strengthen overall pandemic preparedness.

Appendix Additional information about increased pneumonia related emergency department visits, northern Italy.
