# Supplementary material for: Increased Pneumonia-Related Emergency Department Visits, Northern Italy
Source: Emerg Infect Dis. 2025 May;31(5):1057–9. doi: 10.3201/eid3105.241790 (PMC12044229; doi:10.3201/eid3105.241790)
Supplement: Appendix — Additional information about increased pneumonia related emergency department visits, northern Italy. [file 24-1790-Techapp-s1.pdf]

Article DOI: https://doi.org/10.3201/eid3105.241790

EID cannot ensure accessibility for supplementary materials supplied by authors. Readers who have difficulty accessing supplementary content should contact the authors for assistance.

Increased Pneumonia Related Emergency Department Visits, Northern Italy

Appendix

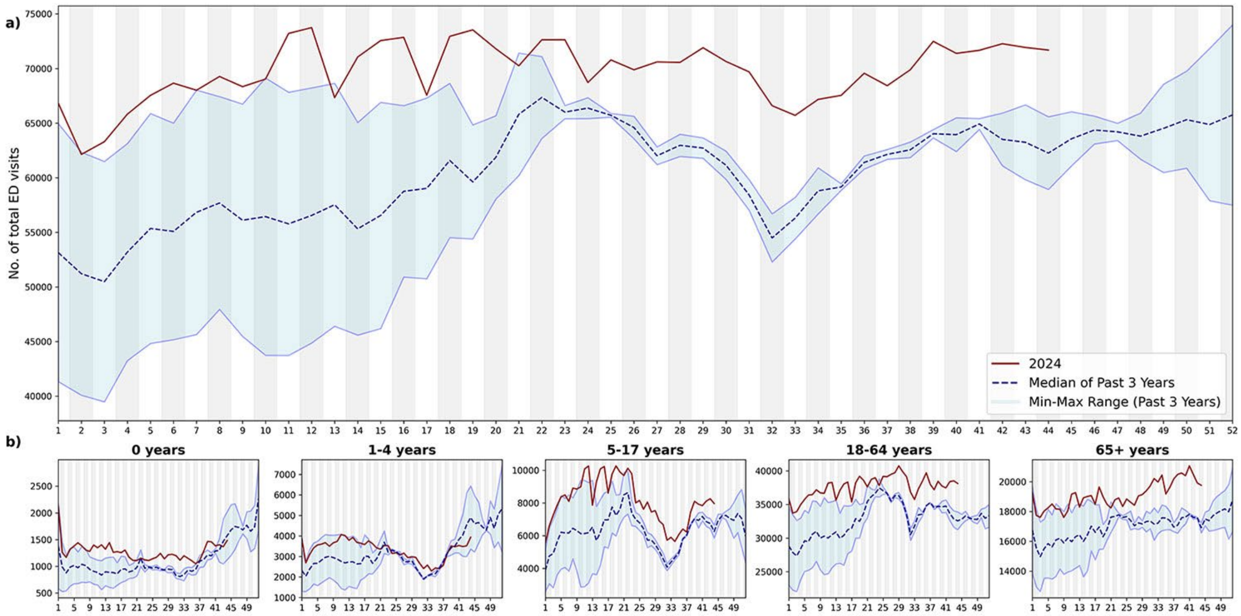

Appendix Figure 1. Weekly number of total emergency department Visits across (a) all and (b) by age group.

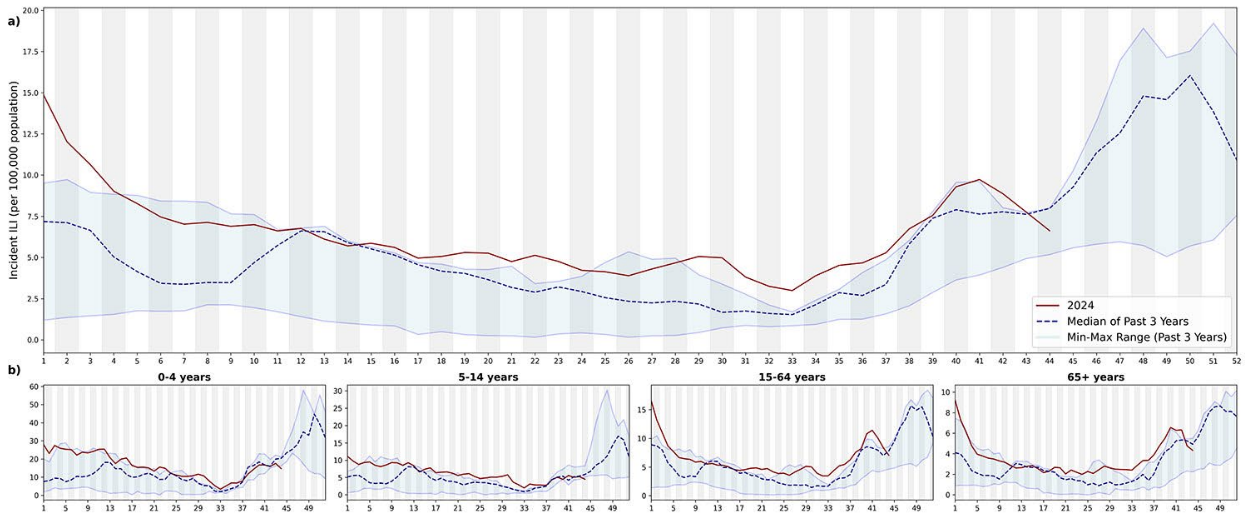

Appendix Figure 2. Weekly number of influenza-like infections reported by General Practitioners and General Pediatricians across (a) all and (b) by age groups.
